# Co-Occurring Heterozygous *CNOT3* and *SMAD6* Truncating Variants: Unusual Presentation and Refinement of the IDDSADF Phenotype

**DOI:** 10.3390/genes12071009

**Published:** 2021-06-30

**Authors:** Manuela Priolo, Francesca Clementina Radio, Simone Pizzi, Letizia Pintomalli, Francesca Pantaleoni, Cecilia Mancini, Viviana Cordeddu, Emilio Africa, Corrado Mammì, Bruno Dallapiccola, Marco Tartaglia

**Affiliations:** 1 Unità di Genetica Medica, Grande Ospedale Metropolitano “Bianchi-Melacrino-Morelli”, 89124 Reggio Calabria, Italy; letizia.pintomalli@libero.it (L.P.); corradomammi@tiscali.it (C.M.); 2 Area di Ricerca Genetica e Malattie Rare, Ospedale Pediatrico Bambino Gesù, IRCCS, 00146 Rome, Italy; fclementina.radio@opbg.net (F.C.R.); simone.pizzi@opbg.net (S.P.); francesca.pantaleoni@opbg.net (F.P.); cecilia.mancini@opbg.net (C.M.); bruno.dallapiccola@opbg.net (B.D.); 3 Dipartimento di Oncologia e Medicina Molecolare, Istituto Superiore di Sanità, 00161 Rome, Italy; viviana.cordeddu@iss.it; 4 UOC di Neuroradiologia, Grande Ospedale Metropolitano “Bianchi-Melacrino-Morelli”, 89124 Reggio Calabria, Italy; emilioafrica@virgilio.it

**Keywords:** IDDSADF, *CNOT3*, facial features profiling, *SMAD6*, aortic coarctation, bicuspid aortic valve, dual molecular diagnosis, exome sequencing

## Abstract

**Objective**, the application of genomic sequencing in clinical practice has allowed us to appreciate the contribution of co-occurring pathogenic variants to complex and unclassified clinical phenotypes. Besides the clinical relevance, these findings have provided evidence of previously unrecognized functional links between genes in the context of developmental processes and physiology. **Patients and Methods**, a 5-year-old patient showing an unclassified phenotype characterized by developmental delay, speech delay, peculiar behavioral features, facial dysmorphism and severe cardiopathy was analyzed by trio-based whole exome sequencing (WES) analysis to identify the genomic events underlying the condition. **Results**, two co-occurring heterozygous truncating variants in *CNOT3* and *SMAD6* were identified. Heterozygous loss-of-function variants in *CNOT3*, encoding a subunit of the CCR4-NOT protein complex, have recently been reported to cause a syndromic condition known as intellectual developmental disorder with speech delay, autism and dysmorphic facies (IDDSADF). Enrichment of rare/private variants in the *SMAD6* gene, encoding a protein negatively controlling transforming growth factor β/bone morphogenetic protein (TGFB/BMP) signaling, has been described in association with a wide spectrum of congenital heart defects. We dissected the contribution of individual variants to the complex clinical manifestations and profiled a previously unappreciated set of facial features and signs characterizing IDDSADF. **Conclusions**, two concomitant truncating variants in *CNOT3* and *SMAD6* are the cause of the combination of features documented in the patient resulting in the unique multisystem neurodevelopmental condition. These findings provide evidence for a functional link between the CCR4-NOT complex and TGFB/BMP signaling in processes controlling cardiac development. Finally, the present revision provides evidence that IDDSADF is characterized by a distinctive facial gestalt.

## 1. Introduction

Proband-parents whole exome sequencing (trio-WES) analysis has substantially improved the chance of obtaining a genetic diagnosis in rare and ultra-rare diseases. In approximately 5% of cases, WES also allows dissection of complex phenotypes as the result of concomitant pathogenic variants involving two or multiple genes (dual molecular diagnoses) [1,2]. Patients with dual diagnoses can be characterized by either qualitatively distinct phenotypes or attenuated/strengthened clinical presentation with respect to the individual diseases depending on the functional link and the role of the genes involved in terms of developmental processes and physiology, eventually resulting in a modulated expression of one or several features [3]. In both cases, a priori discrimination of the contribution of individual pathogenic variants to the complexity of the clinical phenotype is generally a hard task [1]. This is traditionally due to the “common use” of the intuitive principle of parsimony (Occam’s razor), in which the justification based on the simplest assumption (i.e., single genetic cause) is generally preferred in explaining a rare clinical presentation [4]. However, the massive use of WES in selected cohorts of “partially solved” patients (i.e., presentations that only in part are explained by the identified genetic variant, based on the available clinical information for the disorder) has provided evidence that at least one third of cases reported as ‘phenotypic expansion’ actually represents blended phenotypes due to concomitant co-occurring pathogenic variants [5]. The use of genomic sequencing has eventually changed the classical concept of Mendelian inheritance, since alleles with simple Mendelian behavior seem to be an exception rather than the rule in patients with complex phenotypes [3]. Similarly, we appreciate that even in apparently “monogenic diseases” a higher burden of functionally relevant rare variants increases the likelihood of involving modifier alleles able to modulate the phenotypic expression of the driver event [6], either exacerbating or smoothening it [7,8,9]. To add complexity to this landscape, the presence of multiple genetic diseases within a family, each affecting different members with variable modes of inheritance and age-of-onset, might represent an additional confounding element. 

Heterozygous variants in *CNOT3*, encoding a subunit of the CCR4-NOT protein complex, a multimeric regulator with variegated function in mRNA metabolism and gene expression, have recently been reported to cause a developmental delay (DD)/intellectual disability (ID) condition (intellectual developmental disorder with speech delay, autism, and dysmorphic facies (IDDSADF), Mendelian Inheritance in Man (MIM): 618672) [10,11,12]. *CNOT3* variants are largely truncating, which supports haploinsufficiency as the mechanism of disease. 

Small mother against decapentaplegic (SMAD) family member 6 (SMAD6) (MIM: 602931) is a protein negatively controlling signaling elicited by cell surface receptors in response to a number of growth factors belonging to the transforming growth factor β (TGFB) and bone morphogenetic protein (BMP) families, which are secreted proteins regulating cell development and growth [13]. Enrichment of rare/private truncating and MH1/MH2-domain missense variants in *SMAD6* has been reported in association with a wide spectrum of congenital heart defects (CHD). These defects include tetralogy of Fallot (TOF), hypoplastic left heart syndrome, aortic coarctation (CoA) and D-transposition of great arteries (D-TGA), aortic valve disease (AVD, MIM: 614823) with variable degrees of aortic calcifications [14,15,16,17,18,19]. These variants have also been associated with susceptibility to craniosynostosis (MIM: 617439) [20,21], and non-syndromic radioulnar synostosis (RUS, MIM: 179300) [22].

As a paradigmatic example of complex dual diagnosis requiring accurate dissection of clinical features after WES to explain the whole presentation, here we describe a 5-year-old boy affected with a syndromic ID disorder with speech delay, peculiar behavioral phenotype, facial dysmorphism and a severe cardiopathy, who carried two co-occurring pathogenic variants in *CNOT3* and *SMAD6*. We dissect the contribution of individual variants to the complex clinical manifestations of the patient and more accurately characterize the clinical features of IDDSADF by reviewing the previous reports to assess consistent common dysmorphic features and clinical signs. Finally, we also provide evidence for a functional link between the CCR4-NOT complex and TGFB/BMP signaling in processes controlling cardiac development.

## 2. Materials and Methods

### 2.1. Clinical Case

**Figure 1:**
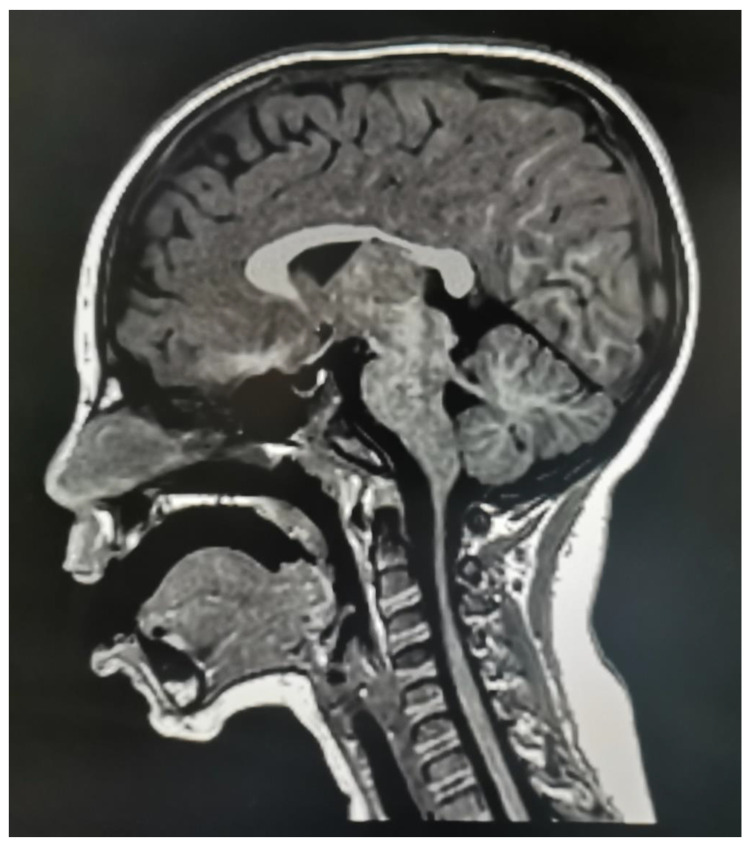
Cerebral magnetic resonance imaging (MRI) scan showing posterior hypoplasia of corpus callosum.

The proband was a 5-year-old boy born at term from apparently non-consanguineous parents after an uneventful pregnancy. Caesarian section was required due to failure in engagement and progression. No exposure to teratogens during pregnancy was reported. Birth weight was 2650 g (5th centile), length 47 cm (10th centile), and head circumference 33 cm (3rd centile). Developmental milestones were normally acquired during the first year, but he presented with language regression soon after 12 months. Language was limited to few words at 4 years. He was diagnosed with aortic stenotic coarctaction (CoA), hypertrophic left ventriculum and bicuspid aortic valve (BAV), surgically treated at 3 years and then pharmacologically controlled afterwards with combined therapy of angiotensin-converting-enzyme (ACE) inhibitor (enalapril) and β blocker (atenolol). In the same period, he was also diagnosed with DD, opposite behavior with emotional outbursts hardly controlled and short span of attention. Suspect of autism spectrum disorder (ASD) was considered, and he was treated with logopedy with limited benefits. At 4 years he presented with facial dysmorphisms, including a prominent forehead, upslanted palpebral fissures, long and thin eyebrows, flat nasal bridge, deep set eyes with periorbital puffiness, low-set anteverted ears with upper folded helix, flabby cheeks with nasolabial sulci prominence, anteverted nares with broad nasal tip, long and smooth philtrum, thin upper lip, and inferior lip prominence with prominent labiomental groove. He also showed a short neck, mild short stature, soft skin and hypermobility of joints. Spontaneous rectal prolapse occurred at 4 years. At our last examination (5 years), he had a weight of 21 Kg (75th centile), height of 108 cm (25th centile) and head circumference of 52 cm (60th centile). The cardiological follow up (5 years), revealed a slight re-coarctation at the former CoA site. A cerebral brain magnetic resonance imaging (MRI) showed hypoplasia of the posterior corpus callosum (Figure 1). In the same period, he was admitted to hospital because of a suspect critical episode that spontaneously resolved.

The proband’s mother is a normally intellectually developed 32-year-old woman. Numerous echocardiograms, thoracic aorta ultrasound evaluations and electrocardiogram (ECG) showed no evidence of cardiopathy, thoracic-abdominal aneurism (TAA) nor anomalies in cardiac conduction. She is currently under cardiac and thoracic aorta follow up with annually repeated ultrasounds.

### 2.2. Methods

The proband was evaluated in the frame of a research program dedicated to undiagnosed patients. The study was conducted in accordance with the declaration of Helsinki principles and after a signed written informed consent was secured. Genomic DNA was extracted from circulating leukocytes. Target regions were captured using the SureSelectAllExon v.7 kit (Agilent, Santa Clara, CA, USA), and sequenced on a NextSeq550 platform (Illumina, San Diego, CA, USA). WES raw data were processed and analyzed using an in-house implemented pipeline previously described [23,24,25] mainly based on the Genome Analysis Toolkit (GATK) Best Practices. Briefly, the GRCh37/hg19 genome assembly was used for reads alignment by means of the Burrows-Wheeler Alignment- Maximal Exact Match (BWA-MEM) tool [26], and HaplotypeCaller (GATK v3.7) for variant calling [27]. Variants/genes functional annotation was made by SnpEff v.4.3 [28] and dbNSFP v.3.5 [29], and various clinical databases (OMIM, ClinVar, HGMD). *In silico* prediction of the impact of variants was performed by Combined Annotation Dependent Depletion (CADD) v.1.4 [30], Mendelian Clinically Applicable Pathogenicity (M-CAP) v.1.0 [31], and Intervar v.2.0.1 [32]. By filtering against an in-house population-matched database (~2500 exomes) and public databases (dbSNP150 and gnomAD V.2.0.1), the analysis was focused on high-quality rare variants affecting coding sequences and adjacent intronic regions. For variant prioritization, we took advantage of an in-house developed scoring system integrating various variant functional annotation (by means of SnpEff), variant clinical impact according to American College of Medical Genetics and Genomics (ACMG) criteria (by means of InterVar), variant functional impact (by means of CADD, M-CAP, S-CAP [33], Spidex [34]), population frequency for the variant in different databases (gnomAD and internal one), gene-tolerance to mutations (Gene Damage Index [GDI] and Residual Variation Intolerance Score [RVIS] tools from dbNSFP v3.5), and genotype-phenotype correlation (by means of Phenolyzer [35]). Sanger sequencing was performed for variant validation and segregation analysis (primers available on request).

Clinical data review of published IDDSADF patients and assessment of available clinical pictures were double blinded performed by three experienced clinical geneticists (P.M., R.F.C. and D.B.).

## 3. Results

Trio-based WES analysis revealed two clinically relevant variants as the molecular causes of the complex phenotype in the proband. A single base insertion in *CNOT3* (c.732dup, p.Ser245GlnfsTer8, NM_014516.4) was identified as a de novo event. A second truncating 19-nucleotide-long deletion in *SMAD6* (c.232_250del, Gln78GlyfsTer41, NM_005585.5) was inherited from the apparently healthy mother. The two variants had previously been reported as pathogenic, causing IDDSADF (rs753475896, ClinVar ID: VCV000694669) [11] and isolated craniosynostosis [20], respectively. No other additional functionally or clinically relevant variant compatible with dominant or recessive (autosomal or X-linked) inheritance model was identified (Supplementary Materials Table S1). Proper coverage excluded occurrence of other variants within the coding exons of the two genes (Supplementary Materials Figure S1).

By reviewing the clinical features associated with pathogenic *CNOT3* and *SMAD6* variants, we suspected a causal role of *CNOT3* haploinsufficiency in the pathogenesis of the peculiar facial features, ID and behavioral anomalies, based on two recently published papers reporting 16 sporadic cases and two families with multiple affected members sharing pathogenic variants in this gene [11,12]. On the other hand, we could ascribe the severe congenital heart defect to the heterozygous truncating variant in *SMAD6*. Loss-of-function *SMAD6* variants have been variably associated with different clinical phenotypes, including cardiac, craniosynostosis and RUS, in the absence of any strict genotype-phenotype correlation. Among the cardiac features, an association between BAV/aortic coartaction and *SMAD6* haploinsufficiency has been established [16,17,18,19].

We reviewed the available published clinical data of IDDSADF to assess consistent dysmorphic features and clinical signs (Table 1). Although previously reports of patients affected with this disorder failed to recognize an obvious facial gestalt [11,12], common dysmorphisms are undoubtedly present. Blind evaluation of the clinical pictures and descriptions of affected individuals by three experienced clinical geneticists evidenced some recurrent facial findings. Affected individuals had prominent forehead (10/21, 47%), flat/high nasal bridge (10/16, 63%) and upslanted palpebral fissures (8/16, 50%). Flabby cheeks with labial sulci prominence were noted in the total of patients (12/12) for whom a published clinical picture was available (pts 1/3/4/7/8/9/11/12/14/16 from Martin et al., 2019; pts II:3 and III:1 family 1 from Meyer et al., 2020). A broad nasal tip (11/21, 52%) with anteverted nares (11/16, 69%) is also a common finding, together with a smooth (11/18, 61%), long philtrum (8/18, 44%) and thin upper lip (11/18, 61%). Prominence of the lower lip with increase of labiomental groove was noted in 75% of patients (9/12) for whom a published clinical picture was available (pts 1/3/4/9/12/14/16 from Martin and colleagues [11]; pts II:3 and III:1 family 1 from Meyer and colleagues [12]). The facial gestalt of the disorder, as described above, is recapitulated well by our proband. Previous reports [11,12] evidenced some additional features (i.e.: low-set eyebrows [11] and triangular face with small chin [12]) in a subset of patients. By reviewing available pictures, we concluded that low set eyebrows are recognizable in three patients from the Martin series (pts 7,11,14) (3/12 available pictures) and that triangular face/small chin actually is not present in available pictures in Meyer series (pts II:3 and III:1 family), thus decided not to include these signs as recurrent.

## 4. Discussion

We identified two co-occurring frameshift variants in *CNOT3* and *SMAD6* as the molecular events likely underlying a complex multisystem phenotype. p.Gln78GlyfsTer41 in SMAD6 is predicted to result in a truncated protein lacking both the MH1 and MH2 domains, which are required for the inhibitory activity of SMAD6 on TGFβ/BMP signaling [13]. Previous studies documented that even more 3′-terminal frameshifts cause a reduced SMAD6 protein level, suggesting either nonsense-mediated decay (NMD) or accelerated protein degradation [21]. Similarly, p.Ser245GlnfsTer8 in CNOT3 is predicted to generate a truncated protein lacking the functional domains downstream the linker region CNOT3-M [36], including the C-terminal region of CNOT3 containing the functionally relevant NOT1 anchor region (NAR) and NOT-box domain. NMD or accelerated protein degradation cannot be ruled out, as well. 

IDDSADF is caused by de novo and inherited variants in *CNOT3* [11,12], which encodes a subunit of a multimeric versatile complex with role in multiple processes controlling gene expression [37,38,39,40,41,42,43]. The spectrum of pathogenic *CNOT3* variants (10 missense and 12 truncating, the present representing the second recurring mutation) and the intolerance for loss-of-function variation (pLI = 1) [44], supports haploinsufficiency as the mechanism of disease. The main IDDSADF clinical features and signs are summarized in Table 1. By reviewing the previous series and comparing them with the present case, we basically confirmed the presence of a recognizable developmental disorder (100%) with speech delay (95%), autism and/or behavioral problems (59%), dysmorphic face and relatively short stature with a median presentation around ≤25th centile (18/22 pts; 82%). The type and severity of DD and learning difficulties is highly variable but invariably present. Speech delay is a constant feature with the exception of one patient [11]. Behavioral problems ranging from pervasive developmental disorder (7/22 pts, 32%) to autism (7/22 pts, 32%) also occur. Muscular hypotonia has been reported in up to 65% of patients. Consistently, our proband showed muscular hypotonia and developed a rectal prolapse at 4 years without any apparently causative trigger. We might speculate on possible pelvic muscle floor hypotonia in the pathogenesis of this unusual finding. Vision impairment, mostly strabismus, is quite frequent (62%). Brain MRI anomalies are common (64%), in particular corpus callosum hypoplasia (5/14 pts; 55% of total brain anomalies), which was also present in our patient. Seizures/electroencephalography (EEG) abnormalities occur in 24% of cases. Our patient manifested a single critical episode without EEG abnormalities. Due to his young age, he is currently under follow up. Based on published data, some recurrent distinctive facial features are recognized, including prominent forehead, flat/high nasal bridge, upslanting palpebral fissures, flabby cheeks with nasolabial sulci prominence, broad nasal tip with anteverted nares, smooth long philtrum, thin upper lip and inferior lip prominence with prominent labiomental groove.

More rarely, *CNOT3* variants have been associated with unilateral/bilateral moyamoya angiopathy (MMA), a vascular trait conferring increased risk to strokes starting in childhood through the fourth decade of life [45]. Among the four reported subjects, the patient with bilateral MMA carried a previously described truncating IDDSADF-causing *CNOT3* variant (p.Gln215*) and presented with ID/ASD, supporting the diagnosis of IDDSADF, even though the lack of detailed clinical description did not allow us to include in our revision. A re-evaluation of our patient’s MRI brain scans excluded signs of MMA. Of note, the remaining three MMA cases did not show ID and/or ASD and carried missense *CNOT3* changes not reported in IDDSADF with a variably functional impact by *in silico* prediction tools or inherited from a normal parent without signs of MMA, thus not supporting their role in this disorder. While available data do not suggest MMA as a major complication of IDDSADF, further studies are required to exclude any link between MMA and IDDSADF.

In the two published IDDSADF series, 2 patients out 21 (9%) had a cardiac anomaly (i.e., perimembranous VSD and abnormal right pulmonary venous return) [11], suggesting that CHD is not a recurrent feature in this disorder. However, our proband presented with a severe heart defect, which was explained by the co-occurring heterozygous *SMAD6* frameshift variant. Smad proteins are intracellular mediators required for regulation of intracellular signal transduction by members of the TGFβ/BMP superfamily [13]. Truncating and missense *SMAD6* variants have been associated with AVD type 2. Incomplete penetrance for cardiovascular features has been reported in *SMAD6* variant carriers (up to 82%) [18]. Variable clinical expressivity within families has also been evidenced, with CoA being a common comorbidity [15,18]. Both cardiac features were present in our patient. While *SMAD6* variants, including the frameshift here reported, have been proposed to contribute to craniosynostosis, in association with a common polymorphism within the *BMP2* locus (rs1884302) [20], this association has not been confirmed in additional craniosynostosis cohorts nor in cardiovascular/RUS patients [15,18,19,21,22]. Available data, however, support a model in which loss-of-function *SMAD6* variants are likely to result in variable phenotypes depending on concomitant genetic or environmental factors, as proved by the recurrence of the same variants in patients affected with either BAV/TAA or craniosynostosis [17,20]. Consistently, our patient had a heterozygous truncating *SMAD6* variant also reported in a patient with isolated craniosynostosis [20], in which the proband’s parent transmitting the *SMAD6* variant was apparently unaffected, as in the present family. Loss-of-function *SMAD6* variants have been found in subjects with late-onset cardiopathies and TAAs and evolutive cardiovascular complications, such as postoperative aneurysm at the former CoA site. In one family, the proband’s father, who was heterozygous for the *SMAD6* variant, deceased at age 80 from diffuse TAA, and had disseminated aortic calcifications [18], which are quite well represented in *SMAD6* variant-positive individuals [19]. In the present family, the proband showed early-onset CoA, which relapsed at the former CoA site 2 years after surgical correction. Although additional studies are required to confirm late-onset complications in these subjects, long-life cardiac monitoring would be highly be recommended. Accordingly, the proband’s mother is currently monitored for cardiac and thoracic aorta complications.

Previous reports in *SMAD6*-related AVD support the idea that pathogenic *SMAD6* variants require a concomitant genetic event to promote adverse clinical outcome. This can be a second hit involving the other *SMAD6* allele, a variant affecting another functionally related gene (TGFB/BMP pathways), or a co-occurring genomic rearrangement (e.g., 15q11.2 BP1-BP2 microdeletion) [18]. In this context, although no direct link between the two proteins is apparent in terms of functional pathways and physical interactions (Supplementary Materials Figure S2), CNOT3 has been identified as a critical regulator in cardiomyocyte differentiation and proliferation in human embryonic stem cells [46]. Silencing of the components of the CCR4-Not complex in *Drosophila* results in myofibrillar disarray and dilated cardiomyopathy [47]. Consistently, heterozygous *Cnot3* knockout (KO) mice display impaired cardiac contractility and increased susceptibility to heart failure [47], while conditional heart and muscle KO *Cnot3* mice are characterized by severe cardiac contractility defects accompanied by long QT intervals and various arrhythmic changes [38]. Thus, it is reasonable to speculate on a possible role of *CNOT3* haploinsufficiency as the “second hit” contributing to the cardiac phenotype associated with the inactivating *SMAD6* variant in the present patient.

## 5. Conclusions

In conclusion, we describe a patient affected with a unique dual molecular diagnosis involving inactivating *CNOT3* and *SMAD6* variants, whose presentation is fully explained by either one (IDDSADF due to *CNOT3* haploinsufficiency) or concomitant events (AVD type 2/CoA due to *CNOT3* and *SMAD6* variants). We also document the occurrence of a distinctive facial gestalt in IDDSADF by defining the recurrent facial features and signs. The present finding suggests a functional link between CCR4-NOT and TGFB/BMP signaling in processes controlling cardiac development. Eventually, we further prove the need for accurate expertise in both clinical and genomic aspects as a key to properly managing patients receiving complex molecular diagnoses. From a clinical perspective, recognizing a multiple molecular cause has several implications for both treatment and the estimation of familial recurrence risk. Multiple variants require careful interpretation of clinical validity by the referring clinician in terms of clinical relevance and proper patient management (i.e., monitoring and follow up), particularly in apparently healthy subjects.

## Figures and Tables

**Table 1:** Clinical signs and facial features in intellectual developmental disorder with speech delay, autism, and dysmorphic facies (IDDSADF).

	Martin et al., 2019 [11]	Meyer et al., 2020 [12]	Present Case	Total
**PATIENTS/FEATURES**				
Sex	6 F/10 M	5 F	M	11F/11M
Type of variant	8MS/8TR	2MS/3TR	TR	10MS/12TR
Low birth weight (≤5th cent)	4/16 SGA *	2/5 SGA	5th cent	
Low birth height (≤5th cent)	NR	4/5 ≤ 5th cent	10th cent	
birth head circumference	NR	5/5 normal range	3rd cent	
Weight	3/15 ≤ 2nd cent	5/5 Normal range	75th cent	
Height	13/16 ≤ 25thcent	4/5 ≤ 10th cent	25th cent	
Head circumference	14/16 cases normal	5/5 normal range	60th cent	
**DEVELOPMENT**				
Developmental delay	16/16	5/5	+	22/22 (100%)
Muscular hypotonia	10/16	2/3	+	13/20 (65%)
Speech delay	15/16	4/4	+	20/21 (95%)
Behavior anomalies (BA)/autism (A)	11/16 (5 BA/7A)	1/5 (BA)	+ (BA)	13/22 (59%)
**EYES**				
Vision	8/15 (4/16 strabism)	4/5 (strabism)	myopia/ astigmatism	13/21 (62%)
**BRAIN MRI ANOMALIES**	7/12	1/1	+	9/14 (64%)
CC hypoplasia	3/12	1/1	+	5/14 (36%) (55%TOT)
Seizures/EEGabnormalities	4/16	1/5	(1 critic episode)	5/21 (24%)
**FACIAL FEATURES**				
Prominent forehead	5/16 °	5/5	+	11/22 (50%)
Flat/high nasal bridge	10/16	nr	+	11/17 (65%)
Upslanted palpebral fissures	8/16 ^#^	nr	+	9/17 (53%)
Low set ears	nr	2/5	+	3/6 (50%)
Flabby cheeks/nasolabial sulci prominence	10/10 ^@^	2/2 ^@^	+	13/13 (100%)
Broad nasal tip	8/16	3/5	+	12/22 (55%)
Anteverted nares	11/16	nr	+	12/17 (71%)
Smooth philtrum	9/16	2/2	+	12/19 (63%)
Long philtrum	6/16	2/2	+	9/19 (47%)
Thin upper lip	9/16	2/2	+	12/19 (63%)
Lower lip prominence with prominent labiomental groove	7/10 ^^^	2/2 ^^^	+	10/13 (77%)

CC, corpus callosum; F, female; M, male; MS, missense; MRI, magnetic resonance imaging; nr, not reported; SGA, small for gestational age; TR, truncating. * Patients 2, 5, 6, 12. ° Patients 2, 3, 6, 9, 15. Data reported in table. Picture available for patients 3 and 9. ^#^ Patients 4, 5, 8, 9, 11, 12, 14, 16. ^@^ Patients 1, 3, 4, 7, 8, 9, 11, 12, 14, 16 from Martin series, and patients II:3 and III:1 (family 1) from Meyer series. Virtually all patients for whom pictures were available had this sign (12/12). The present case had this sign. ^^^ Patients 1, 3, 4, 9, 12, 14, 16 from Martin series, and patients II:3 and III:1 (family 1) from Meyer series. The present case had this sign.

## Data Availability

Data that support the findings of this study are available from the corresponding authors upon request.

## Supplementary Materials

The following are available online at https://www.mdpi.com/2073-4425/12/7/1009/s1, Figure S1: Coverage of the *SMAD6* and *CNOT3* exons, Figure S2: *CNOT3*/*SMAD6* gene network analysis, Table S1: WES statistics and data output.

## Abbreviations

ASD: autism spectrum disorder; AVD, aortic valve disease; BA, behavior anomalies; BAV, bicuspid aortic valve; CHD, congenital heart defects; *CNOT3*, CCR4-NOT protein complex subunit 3; CoA, aortic stenotic coarctaction; IDDSADF, intellectual developmental disorder with speech delay, autism and dysmorphic facies; MH, MAD homology; RUS, non-syndromic radioulnar synostosis; MMA moyamoya angiopathy; MRI, magnetic resonance imaging; NR, not reported, *SMAD6,* small mother against decapentaplegic family member 6; TAA, thoracic aorta aneurism; TGFβ/BMP, transforming growth factor β/bone morphogenetic protein; WES, whole exome sequencing.

## References

[1] Posey J.E., Harel T., Liu P., Pengfel L., Rosenfeld J., James R.A., Akdemir Z.H.C., Walklewitz M., Bi W., Xiao R. (2017). Resolution of disease phenotypes resulting from multilocus genomic variation. N. Engl. J. Med..

[2] Smith E., Blanco K., Sayan S.A., Hunter J.M., Shinde D.N., Wayburn B., Rossi M., Huang J., Stevens C.A., Muss C. (2019). A retrospective review of multiple findings in diagnostic exome sequencing: Half are distinct and half are overlapping diagnoses. Genet. Med..

[3] Papadimitriou S., Gazzo A., Versbraegen N., Nachtegael C., Aerts J., Moreau Y., Van Dooren S., Nowe A., Smits G., Lenaerts T. (2019). Predicting disease-causing variant combinations. Proc. Natl. Acad. Sci. USA.

[4] Balci T.B., Hartley T., Xi Y., Dyment D.A., Beaulieu C.L., Bernier F.P., Dupuis L., Horvath G., Mendoza-Londono R., Prased C. (2017). Debunking Occam’s razor: Diagnosing multiple genetic diseases in families by whole-exome sequencing. Clin. Genet..

[5] Karaca E., Posey J.E., Coban Akdemir Z., Pehlivan D., Harel T., Jhangiani S.N., Bayram Y., Song X., Bahrambeigi V., Yuregir O.O. (2018). Phenotypic expansion illuminates multilocus pathogenic variation. Genet. Med..

[6] Pizzo L., Jensen M., Polyak A., Resenfeld J.A., Mannik K., Krishnan A., McCready E., Pichon O., Le Caignec C., Krishnan A. (2019). Rare variants in the genetic background modulate cognitive and developmental phenotypes inindividuals carrying disease-associated variants. Genet. Med..

[7] Guo T., Chung J.H., Wang T., McDonald-McGinn D.M., Kates W.R., Hawuła W., Coleman K., Zackai E., Emanuel B.S., Morrow B.E. (2015). Histone modifier genes alter conotruncal heart phenotypes in 22q11.2 deletion syndrome. Am. J. Hum. Genet..

[8] Grillo E., Rizzo C.L., Bianciardi L., Bizzarri V., Baldassarri M., Spiga O., Furini S., De Felice C., Signorini C., Leoncini S. (2013). Revealing the complexity of a monogenic disease: Rett syndrome exome sequencing. PLoS ONE.

[9] Schuurs-Hoeijmakers J.H., Oh E.C., Vissers L.E., Swinkels M.E.M., Gilissen C., Willemsen M.A., Holvoet M., Steehouwer M., Veltman J.A., de Vries B.B.A. (2012). Recurrent de novo mutations in PACS1 cause defective cranial-neural-crest migration and define a recognizable intellectual-disability syndrome. Am. J. Hum. Genet..

[10] Deciphering Developmental Disorders Study (2017). Prevalence and architecture of de novo mutations in developmental disorders. Nature.

[11] Martin R., Splitt M., Genevieve D., Aten E., Collins A., de Bie C.I., Faivre L., Foulds N., Giltay J., Ibitoye R. (2019). De novo variants in CNOT3 cause a variable neurodevelopmental disorder. Eur. J. Hum. Genet..

[12] Meyer R., Begemann M., Demuth S., Kraft F., Dey D., Schuler H., Busse S., Hausler M., Zerres K., Kurth I. (2020). Inherited cases of CNOT3-associated intellectual developmental disorder with speech delay, autism, and dysmorphic facies. Clin. Genet..

[13] Miyazawa K., Miyazono K. (2017). Regulation of TGF-β family signaling by inhibitory smads. Cold Spring Harb. Perspect. Biol..

[14] Tan H.L., Glen E., Topf A., Hall D., O’Sullivan J., Sneddon L., Wren C., Avery P., Lewis R.J., ten Dijke P. (2012). Nonsynonymous variants in the SMAD6 gene predispose to congenital cardiovascular malformation. Hum. Mutat..

[15] Jin S.C., Homsy J., Zaidi S., Lu Q., Zen X., Qi H., Chang W., Morton S., De Palma S.R., Halder S. (2017). Contribution of rare inherited and de novo variants in 2,871 congenital heart disease probands. Nat. Genet..

[16] Kloth K., Bierhals T., Johannsen J., Harms F.L., Juusola J., Johnson M.C., Grange D.K., Kutsche K. (2019). Biallelic variants in SMAD6 are associated with a complex cardiovascular phenotype. Hum. Genet..

[17] Gillis E., Kumar A.A., Luyckx I., Preuss C., Cannaerts E., van de Beek G., Wieschendorf B., Alaerts M., Bolar N., Vandeweyer R. (2017). Candidate gene resequencing in a large bicuspid aortic valve-associated thoracic aortic aneurysm cohort: SMAD6 as an important contributor. Front. Physiol..

[18] Luyckx I., MacCarrick G., Kempers M., Meester J., Geryl C., Rombouts O., Peeters N., Claes C., Boeckx N., Sakalihasan N. (2019). Confirmation of the role of pathogenic SMAD6 variants in bicuspid aortic valve-related aortopathy. Eur. J. Hum. Genet..

[19] Park J.E., Park J.S., Jang S.Y., Park S.E., Kim J.W., Ki C.S., Kim D.K. (2019). A novel SMAD6 variant in a patient with severely calcified bicuspid aortic valve and thoracic aortic aneurysm. Mol. Genet. Genom. Med..

[20] Timberlake A.T., Choi J., Zaidi S., Lu Q., Nelson-Williams C., Brooks E.D., Bilguvar K., Tikhonova I., Mane S., Yang J.F. (2016). Two locus inheritance of non-syndromic midline craniosynostosis via rare *SMAD6* and common *BMP2* alleles. eLife.

[21] Calpena E., Cuellar A., Bala K., Swagemakers S.M.A., Koelling N., McGowan S.J., Phipps J.M., Balasubramanian M., Cunningham M.L., Douzgou S. (2020). *SMAD6* variants in craniosynostosis: Genotype and phenotype evaluation. Genet. Med..

[22] Yang Y., Zheng Y., Li W., Li L., Tu M., Zhao L., Mei H., Zhu G., Zhu Y. (2019). *SMAD6* is frequently mutated in nonsyndromic radioulnar synostosis. Genet. Med..

[23] Bauer C.K., Calligari P., Radio F.C., Caputo V., Dentici M.L., Falah N., High F., Pantaleoni F., Barresi S., Ciolfi A. (2018). Mutations in KCNK4 that Affect Gating Cause a Recognizable Neurodevelopmental Syndrome. Am. J. Hum. Genet..

[24] Flex E., Martinelli S., Van Dijck A., Ciolfi A., Cecchetti S., Coluzzi E., Pannone L., Andreoli C., Radio F.C., Pizzi S. (2019). Aberrant Function of the C-terminal Tail of HIST1H1E Accelerates Cellular Senescence and Causes Premature Aging. Am. J. Hum. Genet..

[25] Radio F.C., Pang K., Ciolfi A., Levy M.A., Hernández-García A., Pedace L., Pantaleoni F., Liu Z., de Boer E., Jackson A. (2021). SPEN haploinsufficiency causes a neurodevelopmental disorder overlapping proximal 1p36 deletion syndrome with an episignature of X chromosomes in females. Am. J. Hum. Genet..

[26] Li H. (2013). Aligning sequence reads, clone sequences and assembly contigs with BWA-MEM. arXiv.

[27] Van der Auwera G.A., Carneiro M., Hartl C., Poplin R., del Angel G., Levy-Moonshine A., Jordan T., Shakir K., Roazen D., Thibault J. (2013). From FastQ Data to High-Confidence Variant Calls: The Genome Analysis Toolkit Best Practices Pipeline. Curr. Protoc. Bioinform..

[28] Cingolani P., Platts A., Wang L.L., Coon M., Nguyen T., Wang L., Land S.J., Lu X., Ruden D.M. (2012). A program for annotating and predicting the effects of single nucleotide polymorphisms, SnpEff: SNPs in the genome of Drosophila melanogaster strain w1118, iso-2; iso-3. Fly.

[29] Liu X., Jian X., Boerwinkle E. (2013). dbNSFP v2.0: A database of human nonsynonymous SNVs and their functional predictions and annotations. Hum. Mutat..

[30] Kircher M., Witten D.M., Jain P., O’Roak B.J., Cooper G.M., Shendure J. (2014). A general framework for estimating the relative pathogenicity of human genetic variants. Nat. Genet..

[31] Jagadeesh K., Wenger A., Berger M., Guturu H., Stenson P., Cooper D., Bernstein J., Bejerano G. (2016). M-CAP eliminates a majority of variants with uncertain significance in clinical exomes at high sensitivity. Nat. Genet..

[32] Li Q., Wang K. (2017). InterVar: Clinical Interpretation of Genetic Variants by the 2015 ACMG-AMP Guidelines. Am. J. Hum. Genet..

[33] Jagadeesh K.A., Paggi J.M., Ye J.S., Stenson P.D. (2019). S-CAP extends pathogenicity prediction to genetic variants that affect RNA splicing. Nat. Genet..

[34] Xiong H.Y., Alipanahi B., Lee L.J., Bretschneider H., Merico D., Yuen R.K., Hua Y., Gueroussov S., Najafabadi H.S., Hughes T.R. (2015). RNA splicing. The human splicing code reveals new insights into the genetic determinants of disease. Science.

[35] Yang H., Robinson P., Wang K. (2015). Phenolyzer: Phenotype-based prioritization of candidate genes for human diseases. Nat. Methods.

[36] Boland A., Chen Y., Raisch T., Jonas S., Kuzuoğlu-Öztürk D., Wohlbold L., Weichenrieder O., Izaurralde E. (2013). Structure and assembly of the NOT module of the human CCR4–NOT complex. Nat. Struct. Mol. Biol..

[37] Wahle E., Winkler G.S. (2013). RNA decay machines: Deadenylation by the Ccr4–Not and Pan2–Pan3 complexes. Biochim. Biophys. Acta.

[38] Yamaguchi T., Suzuki T., Sato T., Takahashi A., Watanabe H., Kadowaki A., Natsui M., Inagaki H., Arakawa S., Nakaoka S. (2018). The CCR4-NOT deadenylase complex controls Atg7-dependent cell death and heart function. Sci. Signal..

[39] Kruk J.A., Dutta A., Fu J., Gilmour D.S., Reese J.C. (2011). The multifunctional Ccr4-Not complex directly promotes transcription elongation. Genes Dev..

[40] Webster M.W., Chen Y.H., Stowell J.A.W., Alhusaini N., Sweet T., Graveley B.R., Coller J., Passmore L.A. (2018). mRNA deadenylation is coupled to translation rates by the differential activities of Ccr4-Not nucleases. Mol. Cell.

[41] Collart M.A. (2016). The Ccr4-Not complex is a key regulator of eukaryotic gene expression. Wiley Interdiscip. Rev. RNA.

[42] Collart M.A., Panasenko O.O. (2017). The Ccr4-Not complex: Architecture and structural insights. Subcell. Biochem..

[43] Elmen L., Volpato C.B., Kervadec A., Pineda S., Kalvakuri S., Alayari N.N., Foco L., Pramstaller P.P., Ocorr K., Rossini A. (2020). Silencing of CCR4-NOT complex subunits affects heart structure and function. Dis. Model. Mech..

[44] https://gnomad.broadinstitute.org/.

[45] Pinard A., Guey S., Guo D., Cecchi A.C., Kharas N., Wallace S., Regalado E.S., Hostetler E.M., Sharrief A.Z., Bergametti F. (2020). The pleiotropy associated with de novo variants in *CHD4*, *CNOT3*, and *SETD5* extends to moyamoya angiopathy. Genet. Med..

[46] Zhou B., Liu J., Ren Z., Yao F., Ma J., Song J., Bennet B., Zhen Y., Wang L., Hu G. (2017). Cnot3 enhances human embryonic cardiomyocyte proliferation by promoting cell cycle inhibitor mRNA degradation. Sci. Rep..

[47] Neely G.G., Kuba K., Cammarato A., Isobe K., Amann S., Zhang L., Murata M., Elmén L., Gupta V., Arora S. (2010). A global in vivo Drosophila RNAi screen identifies NOT3 as a conserved regulator of heart function. Cell.

