# Supplementary material for: Co-Occurring Heterozygous CNOT3 and SMAD6 Truncating Variants: Unusual Presentation and Refinement of the IDDSADF Phenotype
Source: Genes (Basel). 2021 Jun 30;12(7):1009. doi: 10.3390/genes12071009 (PMC8303239; doi:10.3390/genes12071009)

SUPPLEMENTAL INFORMATION

Supplemental Table S1. WES statistics and data output.

Supplemental Figure S1. Coverage of the *SMAD6* and *CNOT3* exons.

Supplemental Figure S2. *CNOT3*/*SMAD6* gene network analysis.

Supplemental Table S1. WES statistics and data output.

WES enrichment kit	SureSelectHuman AllExon v7
Sequencing platform	Illumina NextSeq500
Target regions coverage >10x	96%
Target regions coverage >20x	95%
Average depth on target	170x
Total number of high-quality variants	84,347
Variants with effect on CDS or affecting splice sites[1]	14,423
Private, clinically associated and low frequency variants[2]	264
- Putative disease genes (autosomal recessive trait)[3]	-
- Putative disease genes (autosomal dominant trait)[3]	1[4]
- candidate genes	*CNOT3*
- Putative disease genes (X-linked trait)[3]	2[5]
- candidate genes	--
Other clinically relevant gene variants[3]	1[6], *SMAD6*

[1] High-quality (i.e., QUAL>100, according to the *hard-filtering* step from GATK 'best practices') non-synonymous SNVs and INDELs within coding exons and splice regions (-3/+8).

[2] High-quality functionally relevant private/rare variants (<0.001, gnomAD; <0.01, in-house database including ~2500 exomes).

[3] Functional impact assessed by Combined Annotation Dependent Depletion (CADD) v.1.4 (http://cadd.gs.washington.edu/), Mendelian Clinically Applicable Pathogenicity (M-CAP) v.1.0 (http://bejerano.stanford.edu/mcap/) and Intervar (http://wintervar.wglab.org) v2.0.1. Variants predicted as benign or likely benign by Intervar were discarded and only those with CADD score>15 or M-CAP score>0.025 were retained.

[4]*CNOT3* (c.732dup, p.Ser245GlnfsTer8, *de novo*; NM_014516.4)

[5]*SOX3* (c.310G>A, p.Gly104Arg, NM_005634.3), *SLC10A3* (c.1379C>T, p.Thr460Met, NM_001142392.3)

[6]*SMAD6* (c.232_250del, Gln78GlyfsTer41, inherited from the mother; NM_005585.5).

Supplemental Figure S1. Coverage of the *SMAD6* and *CNOT3* exons. The graphs show depth of coverage of reads mapping to the *SMAD6* (top panel) and *CNOT3* (bottom panel) target regions (hg19). Gene structure according to NCBI RefSeq and genomic coordinates are shown. The black line indicates 20x depth.

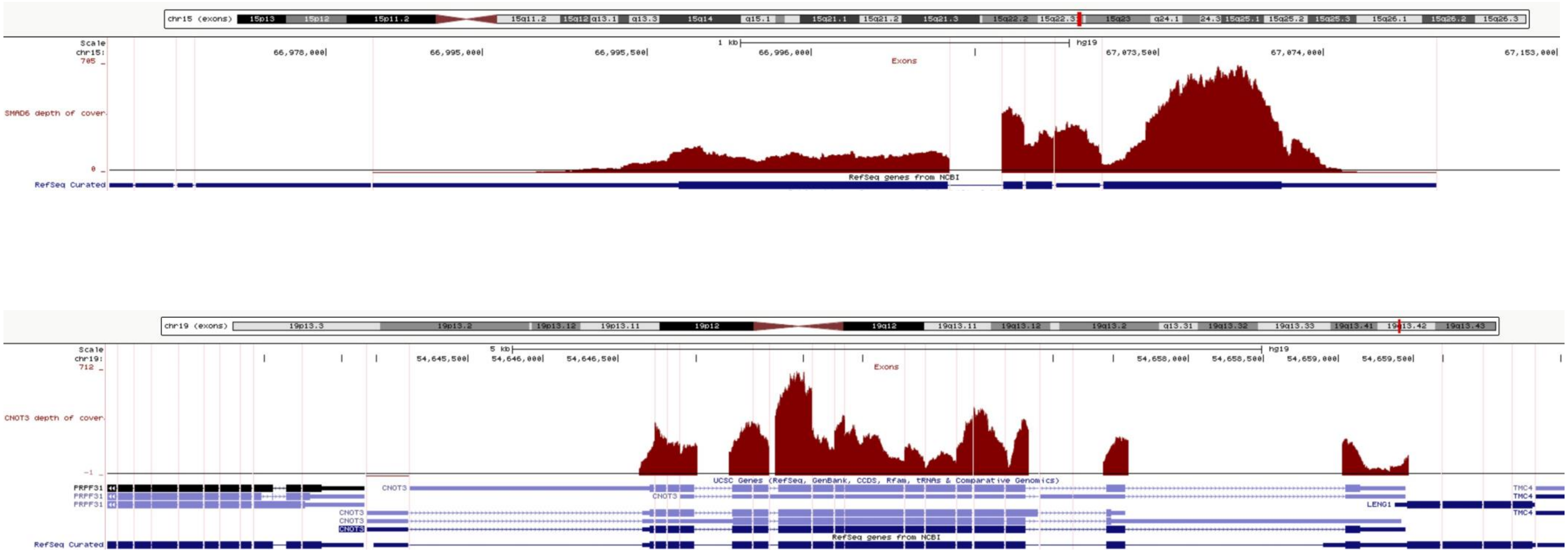

Figure S2. CNOT3/SMAD6 gene network analysis. The plot shows the results of the associations data analysis conducted by geneMANIA (http://pages.genemania.org/). All query genes are given the maximum node size; the size of the nodes for related genes is inversely proportional
to the rank of the gene in a list sorted by the gene score assessed by the tool. The plot highlights independent association networks for the two genes, with the majority of the identified connections occurring within each network.

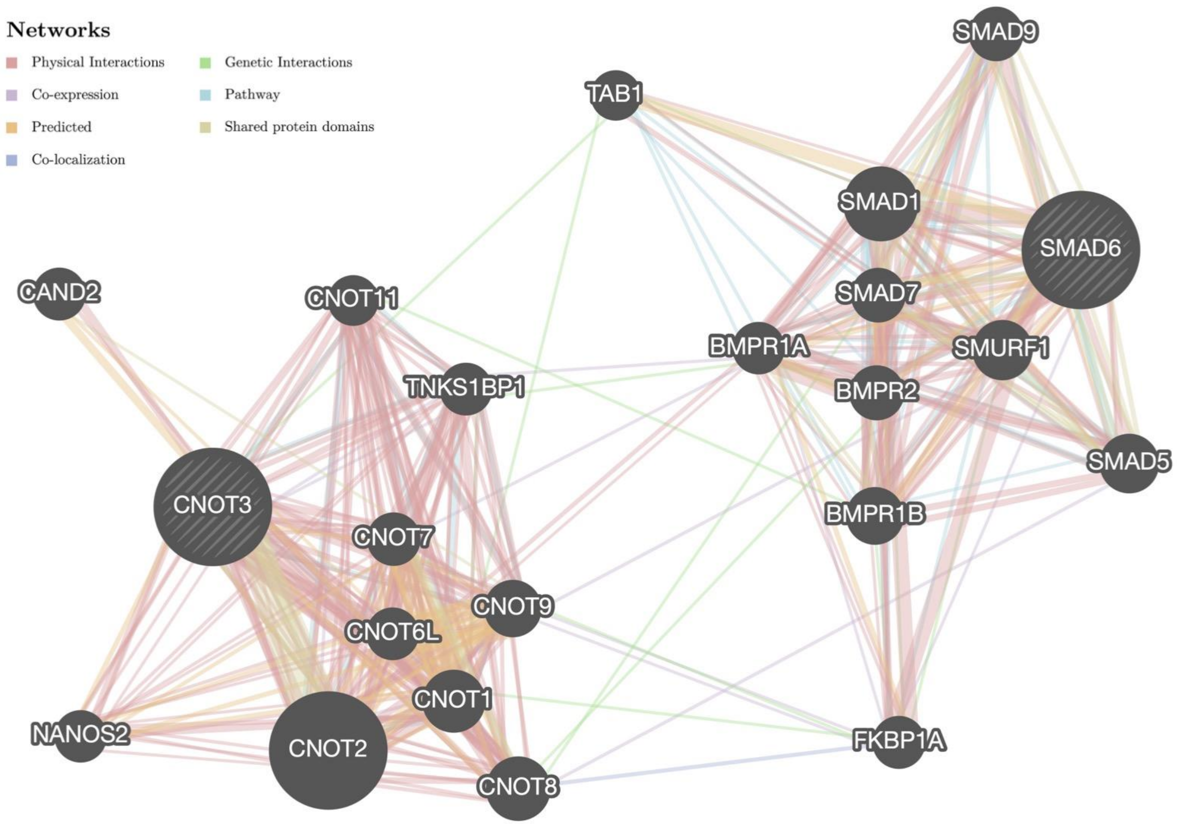

Supplement: Supplementary file 1 [file genes-12-01009-s001.zip › genes-1252311-supplementary.pdf]
